# Recent advances in PTEN signalling axes in cancer

**DOI:** 10.12703/r/9-31

**Published:** 2020-12-23

**Authors:** Jonathan Tak-Sum Chow, Leonardo Salmena

**Affiliations:** 1 Department of Pharmacology and Toxicology, University of Toronto, Toronto, Ontario, Canada; 2 Princess Margaret Cancer Centre, University Health Network, Toronto, Ontario, Canada

**Keywords:** PTEN signalling, nuclear PTEN, PI3K pathway, cancer, tumour suppression

## Abstract

In over two decades since the discovery of phosphatase and tensin homologue deleted on chromosome 10 (PTEN), nearly 18,000 publications have attempted to elucidate its functions and roles in normal physiology and disease. The frequent disruption of PTEN in cancer cells was a strong indication that it had critical roles in tumour suppression. Germline *PTEN* mutations have been identified in patients with heterogeneous tumour syndromic diseases, known as PTEN hamartoma tumour syndrome (PHTS), and in some individuals with autism spectrum disorders (ASD). Today we know that by limiting oncogenic signalling through the phosphoinositide 3-kinase (PI3K) pathway, PTEN governs a number of processes including survival, proliferation, energy metabolism, and cellular architecture. Some of the most exciting recent advances in the understanding of PTEN biology and signalling have revisited its unappreciated roles as a protein phosphatase, identified non-enzymatic scaffold functions, and unravelled its nuclear function. These discoveries are certain to provide a new perspective on its full tumour suppressor potential, and knowledge from this work will lead to new anti-cancer strategies that exploit PTEN biology. In this review, we will highlight some outstanding questions and some of the very latest advances in the understanding of the tumour suppressor PTEN.

## Background

### PTEN the lipid phosphatase

**Figure 1.**
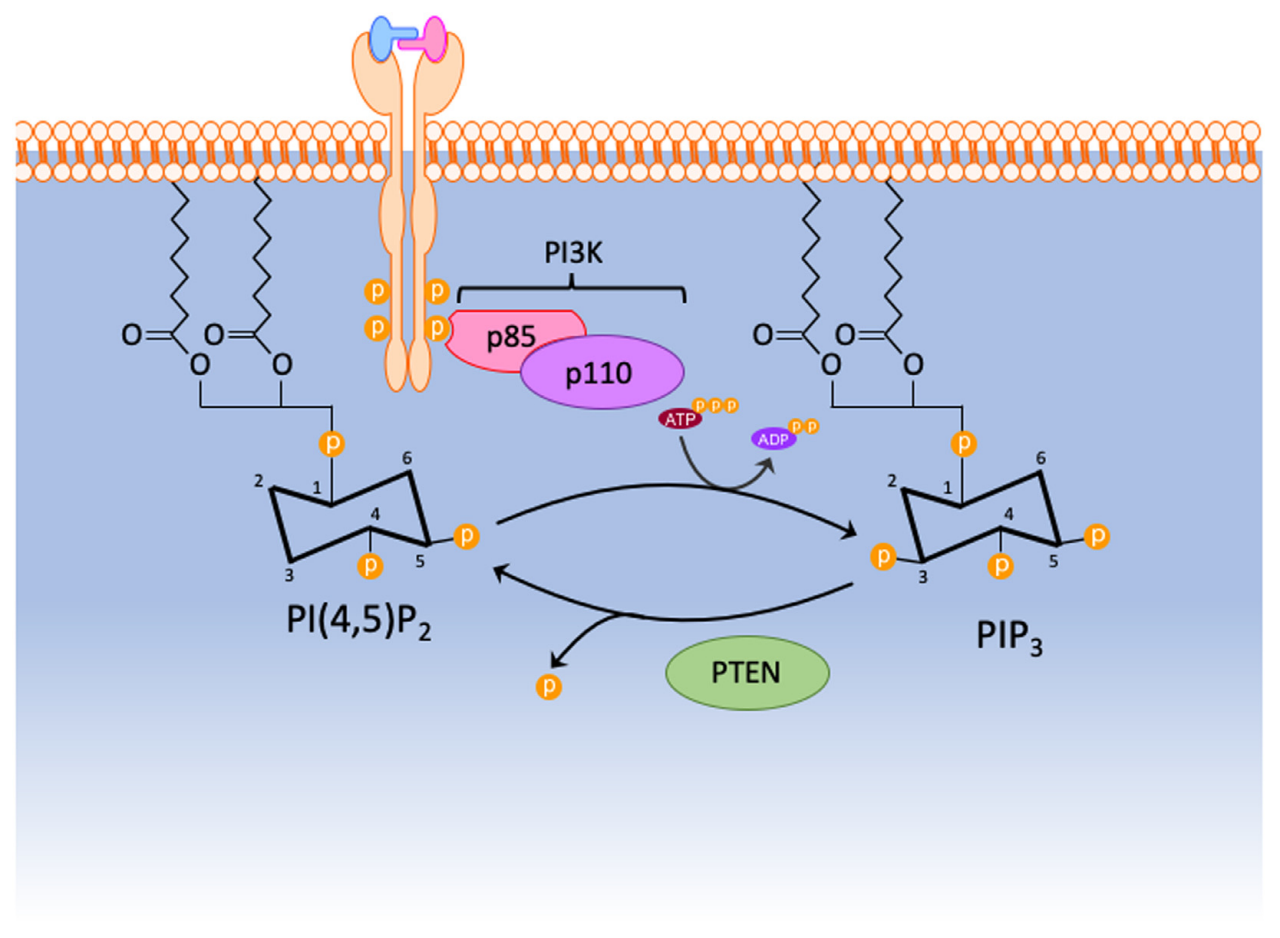
Schematic of PTEN’s lipid phosphatase activity. Briefly, PTEN dephosphorylates the 3 position on the inositol ring of phosphatidylinositol-(3,4,5)-triphosphate (PIP_3_) to generate PI(4,5)P_2_. PI3K, phosphoinositide 3-kinase; PTEN, phosphatase and tensin homologue deleted on chromosome 10.

**Figure 2.**
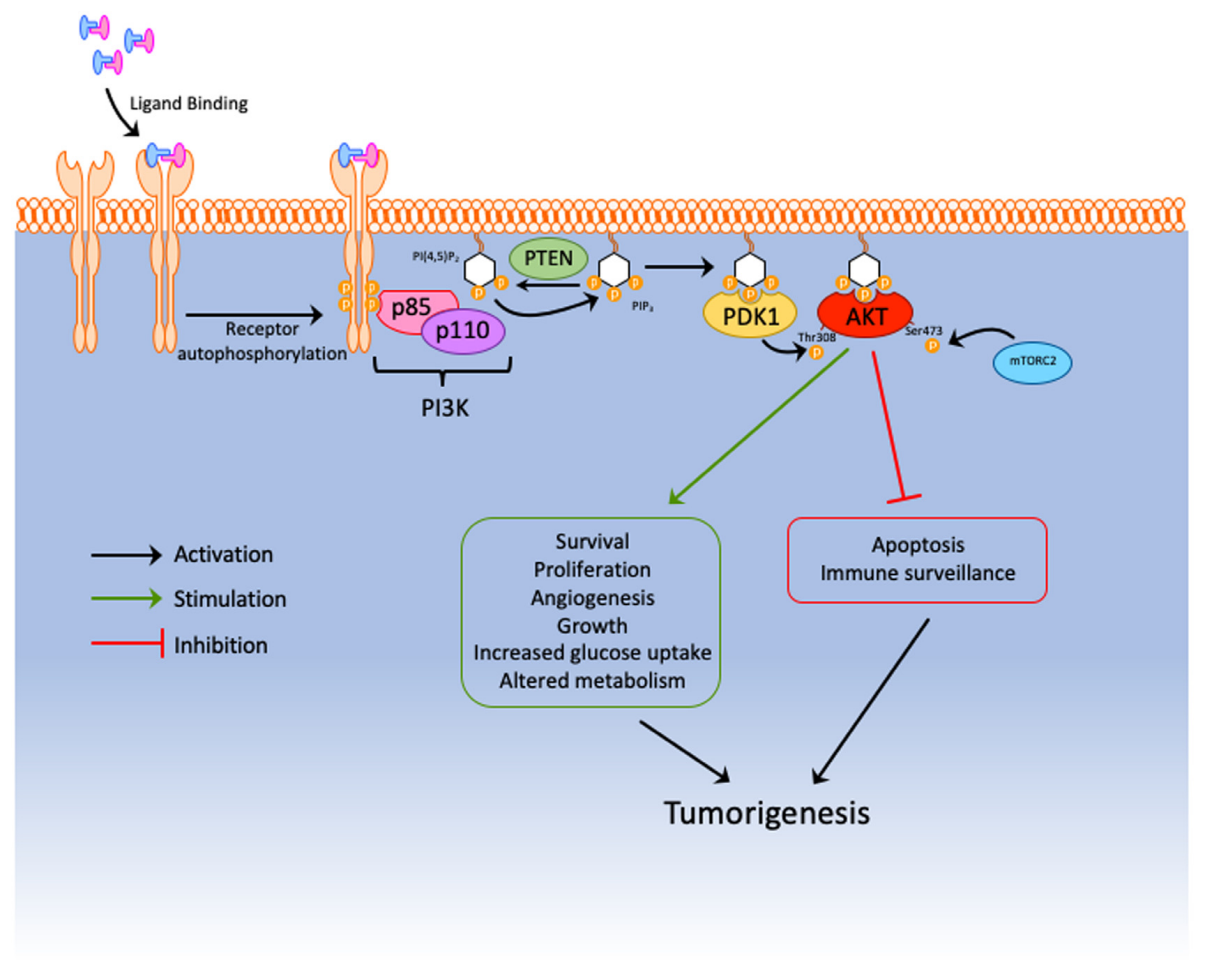
PTEN opposes the PI3K pathway and downstream oncogenic signalling to AKT. By dephosphorylating PIP_3_, PTEN prevents the activation of AKT via PDK1 and thus protects against tumorigenesis. mTORC2, mammalian target of rapamycin complex 2; PDK1, phosphoinositide-dependent protein kinase 1; PI3K, phosphoinositide 3-kinase; PIP_3_, phosphatidylinositol-(3,4,5)-triphosphate; PTEN, phosphatase and tensin homologue deleted on chromosome 10.

Best known as a critical tumour suppressor, phosphatase and tensin homologue deleted on chromosome 10 (PTEN) is a key member of a complex intracellular phosphoinositide signalling network. The canonical function of PTEN is as a lipid phosphatase that dephosphorylates the 3 position on the inositol ring of phosphatidylinositol-(3,4,5)-triphosphate (PIP_3_) to generate PI(4,5)P_2_^1^ (Figure 1). By this mechanism, PTEN opposes signalling of the oncogenic phosphoinositide 3-kinase (PI3K) pathway by limiting the recruitment and activation of AKT at the cell membrane^1,2^ (Figure 2). Loss of PTEN function in cancer cells (through a diversity of mechanisms that are not discussed in this review because of space limitations) almost invariably leads to accumulation of PIP_3_ and associated activation of AKT signalling. Downstream activation of PI3K pathway effectors in cancer are the foremost hallmarks of PTEN loss; however, PTEN loss has also been demonstrated to activate a plethora of pathways including Ras–MAPK, Wnt/β-catenin, Notch, and Hippo pathways through PIP_3_-dependent signals^3–7^. Overall, many other thousands of publications have cemented PTEN as an essential tumour suppressive phosphoinositide phosphatase that controls crucial signalling events and processes including growth, proliferation, survival, and migration^8–12^.

### PTEN the protein phosphatase

Although critically debated since its discovery, PTEN’s protein phosphate activities have been shown to contribute to its tumour suppressive function by an increasing number of studies^13–15^. By using specific mutants of PTEN lacking lipid phosphatase function, an early study concluded that PTEN may block cell migration through a protein phosphatase-mediated function on focal adhesion kinase (FAK) protein^14^. PTEN-mediated G1 cell cycle arrest has also been linked to protein phosphatase-mediated downregulation of cyclin D1^16,17^. Since these first studies, PTEN has been reported to directly dephosphorylate an array of proteins involved in cell motility and migration^18^. Convincing data also point to the PTEN protein as its own substrate in an auto-dephosphorylation mechanism at its C-terminal phosphorylation sites^18^.

The most compelling data on this topic come from the generation of mouse models expressing specific loss-of-function mutations of *Pten*^19,20^. By modelling two cancer-associated *PTEN* mutations at Cys124 and Gly129, Wang *et al*. and Papa *et al.*, respectively,** attempted to dissect the specific roles of Pten catalytic activities^19,20^. *Pten* G129E mutation renders the lipid phosphatase activity inactive whilst sparing protein phosphatase activity^21^, whereas the *Pten* C124S mutation eliminates the essential cysteine in the phosphatase consensus site and renders all Pten phosphatase activity dead^22^. In these studies, homozygosity of either of these mutant alleles was associated with embryonic lethality. In adulthood, heterozygosity of either allele was associated with tumour development similar to observations in *Pten* knockout mice^23,24^. Despite some measurable differences in the spectrum of tumours observed with each specific mutant allele, the results from these studies indicate that the lipid phosphatase function of PTEN is responsible for a majority of the PTEN loss-driven cancer phenotypes. Nevertheless, the abundance of *in vitro* data** supporting a protein phosphatase role for PTEN remains compelling. Further cancer and non-cancer focussed studies on *Pten* G129E and C124S could shed light on protein phosphatase functions. Moreover, the characterisation of the cancer-associated *PTEN* Y138L mutation by Davidson *et al.,* with specific loss of PTEN protein phosphatase activity and retention of lipid phosphatase activity, presents a new tool that should be investigated *in vivo*^25^. Investigation of *Pten* G129E, *Pten* C124S, and *Pten* Y138L mice, among others, will provide critical insight into both the physiological and the pathological roles of the lipid and protein phosphatase functions of PTEN. In addition to being a dual specificity phosphatase for lipid and protein substrates, PTEN can also be dephosphorylated at serine/threonine and tyrosine residues. In sum, the physiological relevance of the protein phosphatase and phosphatase-independent functions of PTEN have yet to be clearly elucidated. However, many excellent tools are available to resolve these questions.

### PTEN the nuclear scaffold protein

A tumour suppressive role for nuclear PTEN has been supported by the discovery of a number of novel functions exerted in the nucleus, most of which are independent of its phosphatase activity. Indeed, a phosphatase-independent function of PTEN in the nucleus was observed to be crucial for chromosome stability^26^. This function was attributed to a role for PTEN in centromere organisation via direct physical association with centromere protein C (CENP-C)^26^. Additionally, nuclear regulation of the cell cycle was linked to direct binding of PTEN to the APC/C-E3-ligase^27^, which facilitated binding to APC, in turn facilitating APC/C and CDH1 interaction to promote the tumour-suppressive activity of the CDH1–APC/C complex^27^. PTEN was also found to physically associate with replication protein A1 (RPA1), which is a subunit of the RPA single-strand DNA-binding protein complex essential for maintaining genomic integrity, to thereby stabilise DNA replication forks and to protect against replication stress^28^. In another study, PTEN was observed to interact with histone H1 via the C-terminal domains of both proteins, leading to the maintenance of chromatin condensation and integrity^29^. PTEN can also physically associate with and dephosphorylate MCM2, a subunit of the MCM2–7 protein complex of the replisome^30,31^, to restrict replication fork progression under replicative stress conditions to prevent DNA strand breaks^32^. Moreover, PTEN has been found to associate with stalled replication forks and recruit Rad51, a protein involved in DNA double-strand break repair, to facilitate stalled replication fork restart^33^. Overall, nuclear functions of PTEN are emerging as critical determinants of the tumour suppressor function of PTEN in disease^34^.

## Recent advances in PTEN regulatory mechanisms

While the PTEN–PI3K axis is well established, there are a plethora of regulatory mechanisms feeding into PTEN and an equal number of downstream mechanisms by which PTEN can function, contributing to the ever-growing complexity of PTEN signalling in cancer. Among these mechanisms, post-translational modifications (PTMs) and protein–protein interactions (PPIs) have been demonstrated to exquisitely control PTEN stability, activity, and localisation. We present a collection of PTEN studies that represent major current findings and highlight exciting directions for future PTEN research.

### PTEN phosphorylation

Novel signalling mechanisms by which inhibitory phosphorylation on the C-terminal tail of PTEN can regulate its tumour suppressive function have been recently uncovered^35,36^. Masson *et al.* previously identified six inhibitory phosphorylation sites in the PTEN C-terminal tail that effectively block both the phosphatase active site and the membrane-binding site of PTEN, where only the unphosphorylated state of PTEN was able to exert its phosphatase activity^37^. Phosphorylation status of T366 and S370 residues in PTEN were also found to influence its catalytic activity. When phosphorylated, these residues occluded the PTEN active site without affecting membrane binding. Notably, partial dephosphorylation at these sites allowed PTEN to act on only select substrates^37^. In supporting work, PDZ Domain Containing 1 (PDZK1) protein was shown to interact with and block phosphorylation of the C-terminal tail of PTEN to allow the PI3K pathway to remain suppressed^38^. Signalling through the PDZK1/PTEN/PI3K axis resulted in reduced growth and proliferation of gastric cancer (GC) cells^38^. Clinically, PDZK1 was low in GC patient specimens and was associated with poor disease prognosis^38^. In another study, the heat shock-like protein Clusterin was shown to increase AKT2 activity and promote the motility of both normal and malignant prostate cells via an inhibitory activity on PTEN-S380 phosphorylation and consequent inactivation of PTEN^39^. Clusterin was also found to specifically reduce the function of the AKT2-specific phosphatase PHLPP1 through miR‐190^39^. In sum, combined suppression of PTEN and PHLPP1 provides evidence for a Clusterin/PTEN/PHLPP1/AKT2 signalling axis involving regulation through miR-190 in prostate cells^39^. Altogether, these studies provide novel insights supporting the importance of C-terminal PTEN phosphorylation as a critical regulatory point of contact on the PTEN protein. Importantly, these findings demonstrate potential therapeutic targets that may mitigate cancer progression, at least in part through the regulation of PTEN phosphorylation at its C-terminus.

### PTEN and ubiquitination

Second only to phosphorylation, PTEN ubiquitination is the most widely studied of all PTMs on PTEN. Indeed, intriguing insights into PTEN-associated cancers have been attributed to mechanisms associated with PTEN ubiquitination^40–47^, a recent example of which is a report demonstrating that the ubiquitin E3 ligase WWP1 can inhibit PTEN function by blocking its dimerisation and membrane recruitment^48^. This study proposed the existence of a putative MYC/WWP1/PTEN oncogenic axis, where WWP1 joins a list of thousands of genes transcriptionally regulated by the pleiotropic MYC oncoprotein^48^. Notably, the study of individuals with germline *WWP1* variants identified gain-of-function effects that support a putative role for *WWP1* as a cancer-susceptibility gene^49^. Finally, a natural compound called indole-3-carbinol was identified as a natural inhibitor of WWP1, thereby identifying a potential therapeutic strategy for cancer prevention and treatment through reactivation of PTEN function^48^.

In another study, the FOXO-regulated deubiquitinase (DUB) USP11 was identified to mediate a PTEN–PI3K autoregulatory loop^50^. This study uncovered that USP11/PTEN signalling integrates with PTEN/PI3K/AKT/FOXO signalling to generate a PTEN feedforward signalling network. Mechanistically, USP11 deubiquitinates PTEN to increase its stability, which promotes the inhibition of PI3K signalling^50^. Conversely, in cells where PI3K and AKT signalling is highly active, AKT-mediated phosphorylation promotes its cytoplasmic sequestration of FOXO. This event reduces USP11 expression and promotes ubiquitin-mediated PTEN degradation to sustain the feedforward PI3K activation that can drive malignant growth. The existence of the PTEN/PI3K/AKT/FOXO/USP11 axis confirms the importance of regulating PTEN stability in cancer.

Two new studies further highlight the importance of PTEN ubiquitination in cancer. First, RPN10, a ubiquitin receptor that is part of the 19S regulatory subunit of the 26S proteasome^51^, was found to promote PTEN ubiquitination and proteasomal degradation in hepatocellular carcinoma (HCC)^52^. Under hypoxic conditions, HIF1α translocation to the nucleus induced transcription of RPN10, leading to the increased degradation of PTEN, elevation of PI3K signalling, and accelerated growth and proliferation of HCC cells^52^. Second, LASP1, an actin-binding protein with roles in cytoskeletal organisation, was found to promote activation of the PI3K pathway and the progression of nasopharyngeal cancer (NPC) by promoting the ubiquitination-mediated degradation of PTEN^53^. The precise mechanism of how LASP1 promotes PTEN ubiquitination still remains elusive^53^. Both the LASP1/PTEN/PI3K/AKT/mTOR and the HIF1α/RPN10/PTEN/PI3K/AKT pathways represent new signalling axes that influence PTEN function and present novel therapeutic avenues.

In sum, these studies add to an increasing body of data demonstrating the diverse consequences of conjugation of monomeric ubiquitin or ubiquitin chains to PTEN including stability, cellular localisation, protein interactions, and catalytic activity^54,55^. The study by Lee *et al.* also exemplifies that agents with modulating effects on ubiquitin ligases and/or deubiquitinases may also be relevant targets for the development of therapies aiming to indirectly enhance PTEN expression^48^.

### PTEN-interacting proteins

Advances in proteomic technologies and bioinformatic approaches for large-scale PPI mapping provide an attractive and emerging approach to identify novel therapeutics^56^. Through such work, novel insights into PTEN-associated PPIs and networks have been uncovered. As the complex interactome of PTEN is methodically unravelled, novel therapeutic approaches can be envisioned through the knowledge these studies provide. As such, PPIs represent potential therapeutic strategies to modulate endogenous levels of PTEN.

Novel studies have uncovered that DMBT1, a tumour suppressor in various cancers, can suppress PI3K pathway signalling through a stabilising interaction with PTEN^57^. In another study, FAM46C protein was demonstrated to inhibit prostate cancer (PCa) growth by promoting PTEN expression levels^58^. FAM46C stabilises PTEN protein by inhibiting ubiquitination to prevent its proteasomal degradation^58^. Sirtuin 6 (SIRT6) was recently reported to interact with PTEN, resulting in higher protein expression levels and lipid phosphatase activity in colon cancer cells^59^. The SIRT6–PTEN interaction was found to promote apoptosis and inhibit cell proliferation *in vitro* through inhibition of PI3K signalling, altogether revealing a novel SIRT6/PTEN/PI3K signalling axis with tumour suppressive capacity^59^.

An interesting comparative study found that the PTEN interactome shared a significant amount of overlap with the interactomes in autism spectrum disorders (ASD) and cancer, suggesting that PTEN is a crucial player in the biology of both diseases^60^. Moreover, this study identified that *PTEN* germline mutations leading to ASD induced a different conformation compared to germline mutations that led to cancer, which may perturb the PTEN interactome in different ways^60^. Given that both ASD and cancer are clinical manifestations of PTEN hamartoma tumour syndrome (PHTS)^61–63^, different germline mutations in PHTS individuals may govern which phenotype occurs by altering the PTEN interactome differently^60^. Overall, like PTMs, PTEN PPIs are emerging as important regulators of PTEN function.

## Recent advances in PI3K-independent functions and beyond

An increasing amount of data suggests that both protein phosphatase activity and phosphatase-independent functions play roles in PTEN-mediated tumour suppression. Peculiarly, this is the case for most of the recently reported PTEN functions in the nucleus, where it has been characterised to have adaptor or scaffold functions. In sum, elucidating novel pathways that involve PTEN signalling will further our understanding and appreciation of PTEN’s role in protecting against tumorigenesis.

### Nuclear PTEN

***Nuclear transport of PTEN.*** A number of experimental and clinical observations have posited that nuclear localisation of PTEN is a contributor to its tumour suppressive functions. Indeed, PTEN is readily detectable in the nucleus of many healthy tissues, whereas nuclear exclusion of PTEN is frequently observed in advanced cancers^64,65^. A recent review on PTEN nuclear function by Ho and colleagues comprehensively described the current state of knowledge^34^. Studies examining PTEN in the nucleus have shed light on how it is transported, retained, or excluded from the nucleus. Mechanisms including monoubiquitination, sumoylation, and direct interactions have also been studied^26,45,47,66–68^. Many such studies utilise mutant PTEN species that harbour non-modifiable residues as clever molecular tools^26,45,47,66–68^. Data suggest that several lysine residues in PTEN have important roles in nuclear translocation mechanisms^45,69^.

In keeping with this theme, a new study has identified that the F-box only protein (FBXO22), a component of the SCF ubiquitin ligase complex, induces ubiquitylation at lysine 221 and degradation of nuclear but not cytoplasmic PTEN^70^. FBXO22 is overexpressed in various cancer types and contributes to the regulation of nuclear PTEN levels in colorectal cancer tissues^70^.

PTEN was also demonstrated to directly interact with the cytoplasmic protein myosin 1b (MYO1B)^71^, which is an actin-binding motor protein^72^. This interaction resulted in nuclear exclusion of PTEN, nuclear AKT activation, and suppression of cell apoptosis^71^. Furthermore, PHTS and ASD-associated germline PTEN Q17E mutant protein was reported to accumulate in the nucleus owing to changes in an N-terminal nuclear localisation sequence. The Q17E mutation and nuclear accumulation of PTEN were posited to have pathogenic effects^73^, illustrating that elevated levels of mutant Q17E PTEN are likely not well tolerated. Interestingly, a cytoplasmic localisation signal (CLS) was previously characterised to be adjacent to Q17 at the PTEN N-terminus, where mutations in this sequence induced PTEN nuclear localisation and subsequently impaired its tumour suppressive activity^74^. Given that Q17E resulted in the nuclear accumulation of PTEN^73^, this CLS could possibly include Q17. However, this study investigated the Q17A mutation and observed cytoplasmic localisation of PTEN^74^, which may suggest that only specific mutations at Q17 induce nuclear localisation.

**Figure 3.**
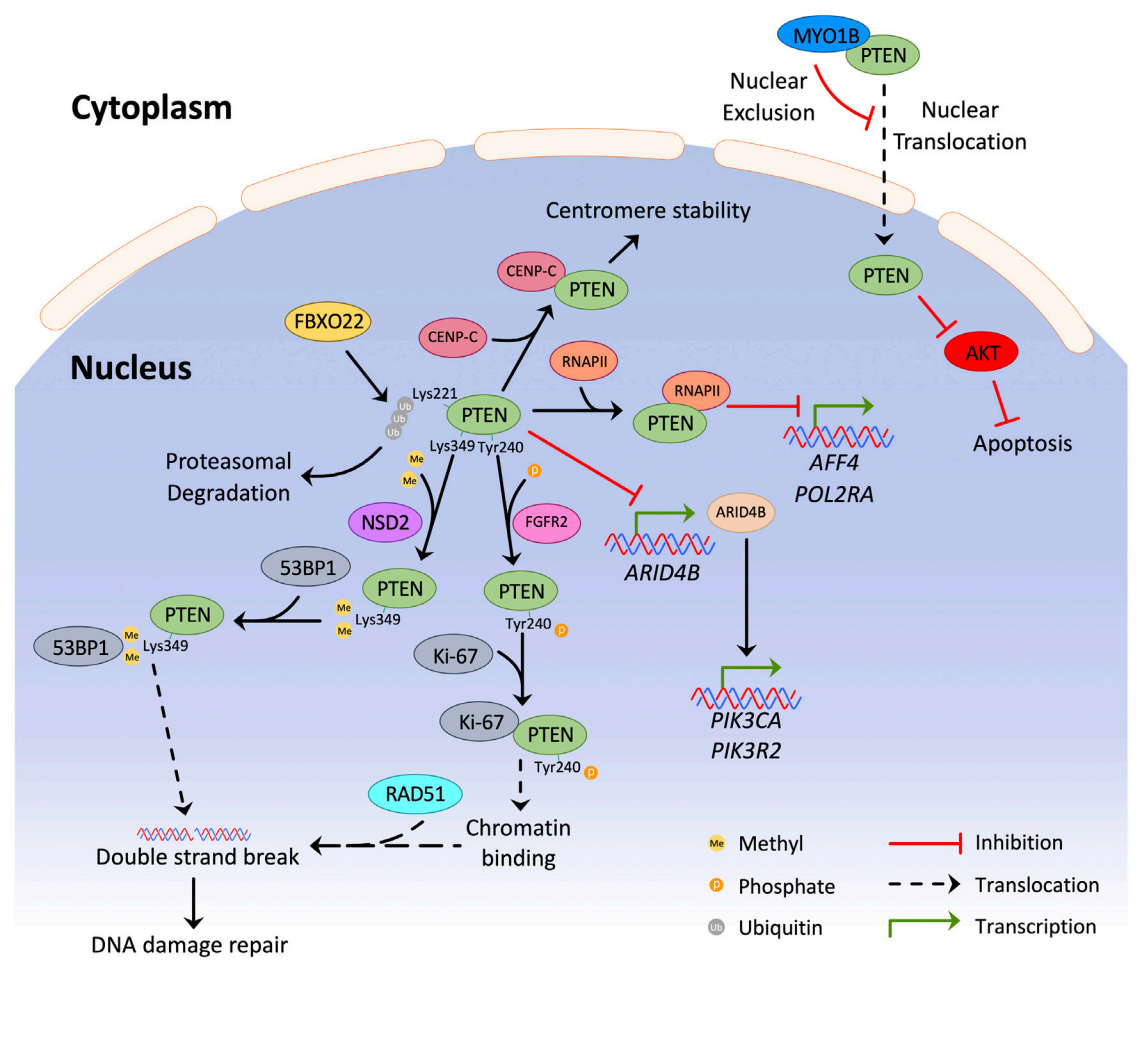
The complexity of PTEN signalling in the nucleus. Schematic representation of the recent advances in PTEN nuclear biology. 53BP1, p53-binding protein 1; ARID4B, AT-rich interaction domain 4B; CENP-C, centromere protein C; FBXO22, F-box only protein; FGFR2, fibroblast growth factor receptor 2; MYO1B, myosin 1b; NSD2, nuclear receptor binding SET domain protein 2; PTEN, phosphatase and tensin homologue deleted on chromosome 10; RNAPII, RNA polymerase II.

***Genome integrity and DNA damage.*** More than localisation, a clear understanding of the importance of PTEN nuclear function remains elusive. Roles for PTEN in DNA damage repair have gained momentum in recent years with studies showing the accumulation of DNA strand breaks in PTEN-deficient cells^66^. More recent contributions to this theme include the discovery that PTEN is a key scaffold protein in DNA repair complexes. One study showed that Nuclear Receptor Binding SET Domain Protein 2 (NSD2)-mediated dimethylation of PTEN promotes 53BP1 interactions and subsequent recruitment to sites of DNA-damage sites^75^. Another study demonstrated that phosphorylation of PTEN on tyrosine 240 by FGFR2 promotes chromatin binding through an interaction with Ki-67, which facilitates the recruitment of RAD51 to promote DNA repair^76^. Figure 3 summarises these novel functions and signalling axes of nuclear PTEN.

***PTEN-associated transcriptional signalling.*** As the repertoire of PTEN functions increases, a number of previously unappreciated roles for PTEN in the regulation of gene expression and processing of RNA transcripts have come to light in the last two years. It is known that AKT signaling plays a critical role in the regulation of pre-mRNA splicing^77^ and PTEN has been shown to modulate G6PD pre-mRNA splicing in an AKT-independent manner^78^. Newer studies add to this small body of data, including an intriguing study where a novel PTEN/ARID4B/PI3K pathway in which PTEN inhibits the expression of ARID4B was characterised. ARID4B is one of several members of the ARID gene family, which are chromatin remodelling factors. PTEN inhibits ARID4B expression and thus prevents the transcriptional activation of ARID4B transcriptional targets *PIK3CA* and *PIK3R2* (PI3K subunits)^79^. This PTEN/ARID4B/PI3K signalling axis identifies a novel player in the PTEN-mediated suppression of the PI3K pathway and provides a new opportunity to design novel therapeutics to target this axis to promote the tumour suppressive functions of PTEN. Furthermore, nuclear PTEN directly interacted with and inhibited RNA polymerase II (RNAPII)-mediated transcription, where it was involved in direct downregulation of critical transcriptional control genes including *AFF4* and *POL2RA*^80^. Similar findings were reported by Abbas *et al.,* where PTEN was found to dephosphorylate the C-terminal domain of RNAPII, leading to its inhibition^81^. They also found that PTEN could modulate genome-wide transcription by redistributing RNAPII across the genome under conditions of metabolic stress^82,83^. Further roles for PTEN in transcriptional modulation were demonstrated in a report where nuclear PTEN interacted with spliceosomal proteins to promote pre-mRNA splicing in a phosphatase-independent manner^84^. PTEN was also found to be dimethylated at Arg159 by PRMT6; this methylation event was demonstrated to be involved in pre-mRNA alternative splicing^85^. Altogether, these studies identify roles for PTEN in global gene regulation and transcript processing that are consistent with previously reported changes in gene expression after loss of PTEN^86,87^. The extensive range of genes that are impacted by PTEN through these mechanisms provides further evidence of a complex role for nuclear PTEN.

### PTEN and other oncogenic signalling pathways

A large body of data demonstrates that PTEN signalling is involved in various cross-talks with other pathways^88^, including Hippo signalling, WNT/β-catenin signalling, and Notch pathways. A large majority of these cross-talk studies demonstrate an indirect association with PTEN through PI3K- and AKT-dependent mechanisms. In this section, we focus most of our discussions on those mechanisms where PTEN is directly linked to other pathways. For instance, the Hippo pathway was linked to the PI3K pathway through PTEN suppression via the induction of miR-29 by the Hippo pathway effector YAP^6^. A more recent study found that the inactivation of the lipid phosphatase activity of PTEN can inhibit the Hippo pathway by promoting the nuclear translocation of YAP and TAZ in GC. Hippo pathway inhibition allows oncogenic transcriptional programs to be induced^89^. These findings suggest that the tumorigenic effect of PTEN inactivation in GC is twofold, as Hippo inactivation is synergistic with the established derepression of PI3K signalling downstream of PTEN inactivation^89^.

Similarly, a large number of studies support that PI3K–AKT and WNT/β-catenin signalling pathways are highly connected. However, a new study highlights a direct interaction of PTEN with β-catenin and Wnt signalling^90^. This study investigated the role of CREB-binding protein (CBP)–β-catenin signalling on both the expression of the stem cell antigen CD133 and the PP2A–PTEN pathway in tumour-initiating cells (TICs) in liver cancer. CBP–β-catenin signalling regulated the levels of C-terminal PTEN phosphorylation in TICs and promoted stemness via CD133 induction. Overall, WNT/β-catenin was demonstrated to control PTEN phosphorylation via a PP2A-dependent mechanism^90^. This study provides a novel link between the two highly oncogenic PI3K and WNT/β-catenin pathways directly through PTEN in the form of a novel CBP/β-catenin/PP2A/PTEN/PI3K/AKT axis^90^.

PTEN and Notch have also been demonstrated to cross-talk extensively, mainly through PI3K- and AKT-dependent mechanisms. However, the evidence for direct interactions between PTEN and Notch signalling make up only a minority of those studies. In one of these studies, Baker *et al.* reported that Notch1 can mediate transcriptional suppression of *PTEN*, resulting in the derepression of PI3K signalling and development of trastuzumab resistance^91^. This study was the first to link the Ras–MAPK and PI3K pathways through Notch1 transcriptional suppression of *PTEN*^91^. Furthermore, the known cancer/testis antigen Plac1 was reported to interact with Furin, a proprotein processing enzyme^92^, to degrade Notch1 into Notch1 intracellular domain (NICD) fragments that undergo nuclear translocation to suppress *PTEN* transcription^93^, forming a Plac1/Furin/Notch1/NICD/PTEN signalling mechanism that results in transcriptional repression of PTEN and allows for the hyperactivation of AKT signalling in breast cancer (BC) cells^93^. Perhaps developing a small molecule to stabilise PPIs in the ASXL1–BAP1 complex could elevate the expression of PTEN and thereby tumour suppressive activity. Conversely, inhibiting the interaction of Plac1 with Furin could derepress PTEN expression. PTEN was also implicated in regulating epithelial–mesenchymal transition (EMT) and metastasis in tongue squamous cell carcinoma through a Numb/Notch1/RBP-Jκ/PTEN/p-FAK/EMT axis^94^. Numb inhibits Notch1, leading to the downregulation of RBP-Jκ^94^, which upregulates PTEN and anti-EMT effectors, leading to the downregulation of p-FAK and pro-EMT effectors^94^. However, the precise mechanisms remain elusive. Is the upregulation of PTEN due to increased transcription or reduced degradation? How does PTEN affect p-FAK levels? In spite of this, this report suggests yet another signalling axis in which PTEN is implicated.

**Figure 4.**
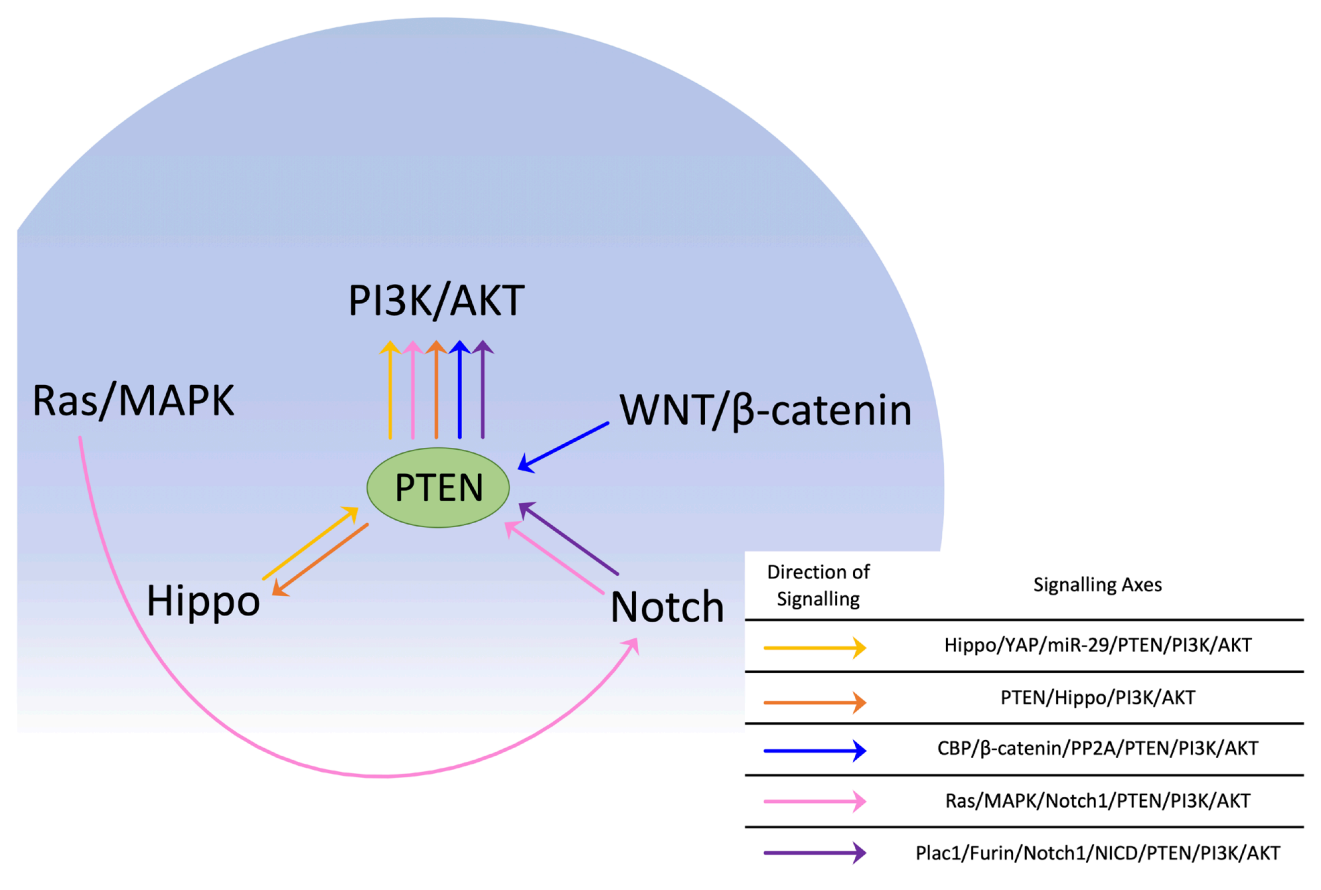
PTEN signalling in other major oncogenic pathways. All of the major oncogenic pathways here signal through PTEN to affect the PI3K pathway. CBP, CREB-binding protein; MAPK, mitogen-activated protein kinase; miR-29, microRNA 29; NICD, Notch1 intracellular domain; PI3K, phosphoinositide 3-kinase; PTEN, phosphatase and tensin homologue deleted on chromosome 10; YAP, Yes-associated protein.

Overall, each of these studies highlight the importance of PTEN signalling in protecting against tumorigenesis and build upon existing bodies of work on the complex crosstalk between PTEN signalling and other pathways. A further understanding of PTEN crosstalk with Hippo, WNT, and Notch signalling (Figure 4) and other signalling pathways in cancer will provide critical insights into an understanding of cancer development as well as novel therapeutic strategies and resistance pathways frequently observed in cancer relapse.

### PTEN metabolic signalling

Metabolic reprogramming in cells is one of the hallmarks of cancer as described by Hanahan and Weinberg^95^. The Warburg effect is one of the most notable metabolic changes that takes place in cancerous cells, where cells become increasingly reliant on glycolysis compared to the more-efficient citric acid cycle^96–98^. In recent years, PTEN has been shown to be involved in the regulation of glycolysis in cancer cells; its loss or inactivation allows cells to become “Warburg-like” and become reliant on glycolysis, consequently making them more aggressive and resistant to chemotherapy. In a study by Qian *et al.*, the protein phosphatase activity of PTEN was linked to metabolic changes that occur in tumorigenesis^99^. It was reported that PTEN could dephosphorylate PGK1, a glycolytic enzyme and protein kinase with a tumorigenic role in glioblastoma^99^. Dephosphorylation of PGK1 by PTEN was found to inhibit its activity, downstream glycolytic functions, and glioblastoma cell proliferation^99^, thereby presenting another mechanism in which PTEN functions as a tumour suppressor. Another role for PTEN in metabolic processes was reported in a study linking it to pyruvate dehydrogenase kinase 1 (PDHK1)^100^. In this study, PTEN was observed to dephosphorylate the NF-κB-activating protein (NKAP) and limit NF-κB activity and downstream transcriptional changes of target genes including *PDHK1*^100^. PTEN and PDHK1 were observed to have a synthetic-lethal relationship, as loss of PTEN and upregulation of PDHK1 in cells induced glycolysis and a dependency on PDHK1^100^. This was supported by observations that PTEN-deficient tumours have elevated PDHK1 levels, which is a biomarker for poor survival^100^. These data point to a potential PTEN/NKAP/NF-κB/PDHK1/glycolysis signalling axis that could potentially be targeted in PTEN-deficient cancers^100^.

In small-cell lung cancer (SCLC) cells, PTEN is targeted and suppressed by miR-214, which subsequently leaves the PI3K/AKT/mTOR pathway unopposed^101^. This was found to signal to hexokinase 2 (HK2) and pyruvate kinase isozyme 2 (PKM2), resulting in the upregulation of glycolysis and proliferation of SCLC cells^101^. Furthermore, inhibition of miR-214 resulted in the elevation of PTEN and downregulation of the PI3K/AKT/mTOR pathway and reversed the effects on glycolysis and proliferation^101^. This suggests that miR-214 and PTEN can signal onto HK2/PKM2 via the PI3K pathway in SCLC cells that regulates glycolysis and proliferation^101^. PTEN was also found to be involved in regulating glycolysis in refractory acute myeloid leukaemia (AML) cells, leading to the development of chemotherapy resistance^102^. In refractory AML cells, PTEN was depleted and phosphorylated AKT was increased compared to non-refractory cells^102^. Moreover, these changes in the PTEN/PI3K/AKT pathway were associated with increased glucose transporter 1 (GLUT1) and HK2 expression as well as lactate production^102^. Inhibition of AKT activity not only decreased proliferation and glycolysis in refractory AML cells but also sensitised these cells to chemotherapy^102^. The data from this study suggest that in refractory AML cells, depletion of PTEN and the unopposed hyperactivity of AKT result in the upregulation of glycolysis and subsequently confer resistance to chemotherapy^102^.

These studies provide more evidence that links PTEN to the regulation of glycolysis in cells. Indeed, suppressing glycolysis appears to be a major endpoint of PTEN tumour suppressive signalling. As the role of PTEN in glycolysis continues to expand, so will the number of possible signalling axes by which PTEN can regulate glycolysis. These new axes can then serve as potential targets in PTEN-deficient cancers that rely on glycolysis for tumorigenesis.

A major clinical challenge in PHTS is predicting which of these clinical manifestations individuals will develop^63^. Given that PTEN signalling has a role in metabolic reprogramming, particularly in glycolysis^99,100^ as we have described, it is intriguing that various tricarboxylic acid (TCA) cycle metabolites were found to be associated with various clinical manifestations of PHTS^103^. This metabolomic study identified that increased isocitrate and reduced citrate levels in PHTS individuals were associated with the development of BC^103^. Fumarate was also identified as a metabolite that was decreased in PHTS individuals who developed ASD compared to those who developed cancer^103^. The differential levels of these TCA metabolites and their association with clinical manifestations of PHTS^103^ could serve as a basis for the future development of prognostic metabolic biomarkers that could help predict the clinical progression of PHTS individuals.

### PTEN isoforms

**Figure 5.**
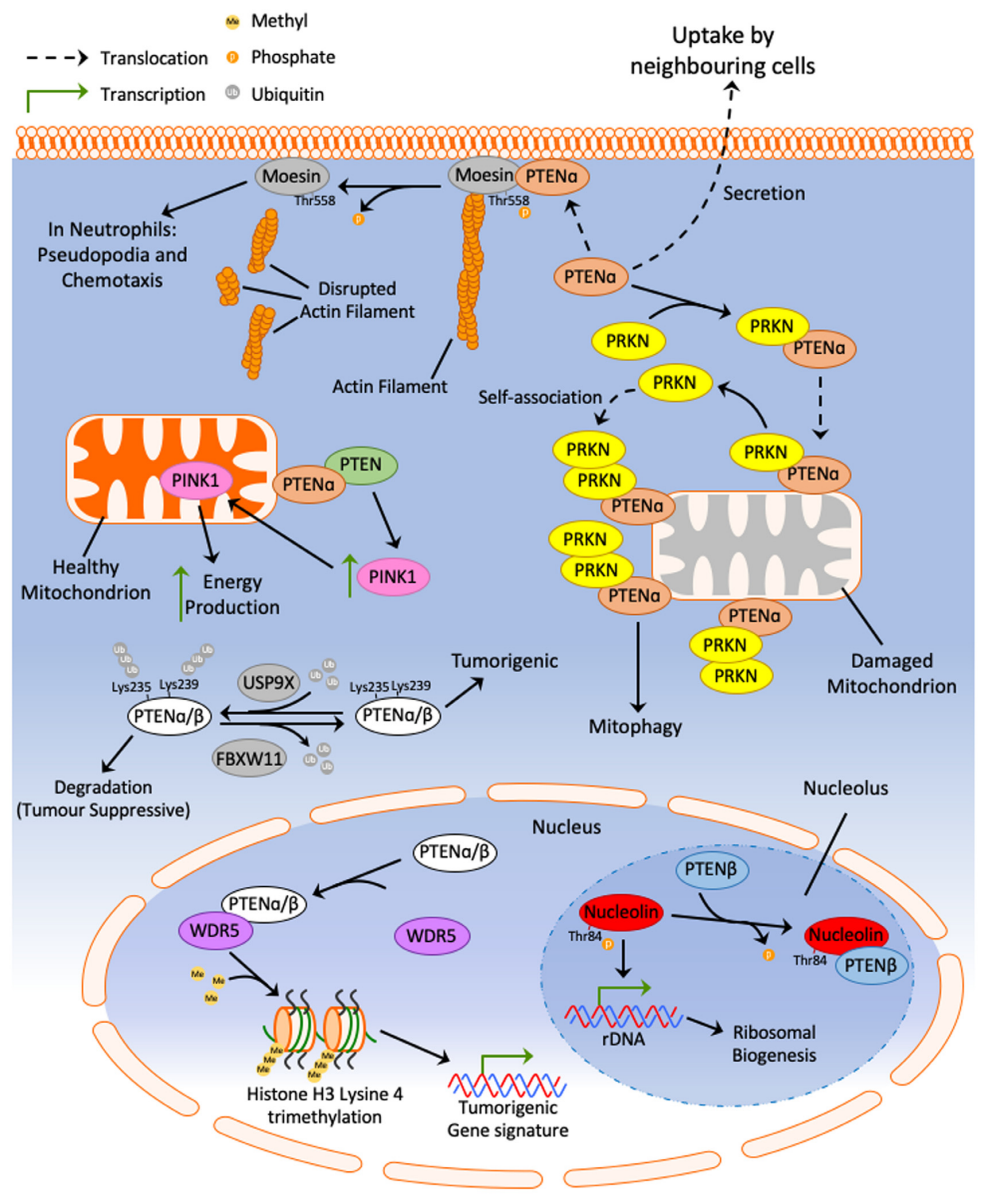
PTEN isoform signalling. Schematic representation of signalling axes involving the PTENα and PTENβ isoforms. Interestingly, PTENα/β appear to have tumour promoting functions that are in contrast to canonical PTEN. FBXW11, F-box/WD repeat-containing protein 11; PTEN, phosphatase and tensin homologue deleted on chromosome 10; PRKN, parkin RBR E3 ubiquitin protein ligase; USP9X, ubiquitin-specific peptidase 9, X-linked; WDR5, WD repeat-containing protein 5.

Several groups have identified alternative translational start sites upstream of the canonical PTEN start codon, resulting in the production of PTEN isoforms with an extended N-terminus^104–106^. To date, only two isoforms have been described: PTENα (or PTEN-Long)^104,105^ and PTENβ^106^. PTEN isoforms including PTENα and PTENβ have been reported to function both in and beyond the PI3K pathway, adding more complexity to the field of PTEN signalling biology (Figure 5).

Initial characterisation of PTENα revealed that this isoform is membrane permeable, is secreted from cells, and can be taken up by neighbouring cells^104^. Indeed, exogenous PTENα was identified to oppose the PI3K pathway in the receiving cells and induced *in vitro* and *in vivo* cell death^104^. These data present a potential approach to restoring PTEN levels in deficient cells that could be explored in future studies^104^. The discovery of the PTENα isoform was subsequently confirmed by Liang *et al.* using mass spectrometry, where it was revealed to be co-localised with canonical PTEN at the mitochondria, suggesting a role in mitochondrial signalling^105^. Colocalisation of PTEN/PTENα promoted the function of PINK1, a mitochondrial-target kinase, and subsequently promoted energy production^105^. PTENα was also shown to play a role in regulating mitophagy through a direct interaction with the mitophagy initiator protein PRKN^107^. PTENα promotes PRKN self-association at the mitochondria in a PTENα phosphatase-independent manner^107^. PTENα/PRKN signalling in mitophagy was supported by evidence demonstrating that the PTENα–PRKN interaction was stronger when mitochondria were damaged and depolarised^107^. PTENα was also reported to regulate neutrophil morphology and chemotaxis through direct binding and dephosphorylation of Thr558 on moesin, a membrane cross-linking protein^108^. Moesin dephosphorylation disrupts actin filaments that are associated with the plasma membrane and results in morphologic changes in neutrophil pseudopodia that are required during chemotaxis^108^. This evidence suggests a role for PTENα, its protein phosphatase activity, and its signalling at the plasma membrane in the regulation of neutrophil morphology and chemotaxis.

PTENβ was more recently identified and has a longer N-terminus than both PTENα and canonical PTEN^106^. Liang *et al.* characterised the localisation of PTENβ at the nucleolus, where it interacts with and dephosphorylates Thr84 on nucleolin^106^. Interaction of PTENβ with nucleolin, a nucleolar protein that is essential in ribosomal biogenesis^109,110^, points to a role in ribosomes and translation^106^. Indeed, PTENβ overexpression was found to regulate rDNA transcription, and inhibiting PTENβ results in the promotion^106^ of ribosomal biogenesis. It was concluded that PTENβ regulates cell proliferation through regulating ribosomal biogenesis; however, an exact signalling mechanism has not been characterised and requires future study.

Given the renowned and classical role of canonical PTEN in tumour suppression, it is plausible to hypothesise that PTENα/β have similar tumour suppressive functions. However, Shen *et al*. demonstrated that PTENα/β isoforms may also be tumour promoting in specific contexts, in contrast to canonical PTEN^111^. Mechanistically, the isoforms were able to promote tumorigenesis by interacting with WDR5 and activating trimethylation of histone H3 lysine 4 (H3K4), which could maintain the expression of a tumour-promoting gene signature^111^. PTENα and PTENβ were also observed to be regulated by ubiquitin-specific peptidase 9, X-linked (USP9X), and F-box/WD repeat-containing protein 11 (FBXW11) through interactions with lysine residues on their extended N-terminal regions^111^. This study presents intriguing first evidence of a contrasting role for PTEN isoforms in the tumorigenic process^111^. Future studies are required to confirm these newly identified functions. Overall, the evidence presented from this study points to a more complex signalling network of PTEN and its isoforms than previously envisioned and raises questions about the established tumour suppressive role of PTEN.

## Future directions and conclusion

It is evident that there is still much to learn about PTEN, as shown by the continuous high pace of discovery. As technological approaches continue to advance, the ability to measure, monitor, detect, visualise, and experimentally manipulate PTEN *in vitro* and *in vivo* brings forth the understanding of novel features of this extraordinary gene and protein. While this review mainly focused on PTEN signalling in cancer, PTEN signalling has been implicated in a variety of other diseases such as PHTS^61–63^, autoimmunity and immunological functions^112^, and other neurodevelopmental disorders^113^; future studies should be aimed at further understanding the role of PTEN signalling in these contexts and how it relates to its renowned function in cancer. Novel PTEN-linked signalling axes revealed by new studies present additional novel approaches for targeting the PTEN pathway for a wide range of diseases, both in and beyond cancer.
